# Variability of Basal Rate Profiles in Insulin Pump Therapy and Association with Complications in Type 1 Diabetes Mellitus

**DOI:** 10.1371/journal.pone.0150604

**Published:** 2016-03-03

**Authors:** Markus Laimer, Andreas Melmer, Julia K. Mader, Ingrid Schütz-Fuhrmann, Heide-Rose Engels, Gabriele Götz, Martin Pfeifer, Julia M. Hermann, Christoph Stettler, Reinhard W. Holl

**Affiliations:** 1 Department of Endocrinology, Diabetology and Clinical Nutrition, University Hospital Bern, Bern, Switzerland; 2 Department of Internal Medicine I, Medical University of Innsbruck, Innsbruck, Austria; 3 Department of Endocrinology and Metabolism, Medical University of Graz, Graz, Austria; 4 Medical Department of Metabolic Disease and Nephrology, Hospital Hitzing, Vienna, Austria; 5 Vivantes Clinics Hellersdorf, Berlin, Germany; 6 Diabetes Centre Nürtingen, Clinics Esslingen, Baden-Württemberg, Germany; 7 Department of Internal Medicine, Hospital Tettnang, Baden-Württemberg, Germany; 8 Institute of Epidemiology and Medical Biometry, ZIBMT, German Center for Diabetes Research (DZD), University of Ulm, Ulm, Germany; Baylor College of Medicine, UNITED STATES

## Abstract

**Background:**

Traditionally, basal rate profiles in continuous subcutaneous insulin infusion therapy are individually adapted to cover expected insulin requirements. However, whether this approach is indeed superior to a more constant BR profile has not been assessed so far. This study analysed the associations between variability of BR profiles and acute and chronic complications in adult type 1 diabetes mellitus.

**Materials and Methods:**

BR profiles of 3118 female and 2427 male patients from the “Diabetes-Patienten-Verlaufsdokumentation” registry from Germany and Austria were analysed. Acute and chronic complications were recorded 6 months prior and after the most recently documented basal rate. The “variability index” was calculated as variation of basal rate intervals in percent and describes the excursions of the basal rate intervals from the median basal rate.

**Results:**

The variability Index correlated positively with severe hypoglycemia (r = .06; p<0.001), hypoglycemic coma (r = .05; p = 0.002), and microalbuminuria (r = 0.05; p = 0.006). In addition, a higher variability index was associated with higher frequency of diabetic ketoacidosis (r = .04; p = 0.029) in male adult patients. Logistic regression analysis adjusted for age, gender, duration of disease and total basal insulin confirmed significant correlations of the variability index with severe hypoglycemia (β = 0.013; p<0.001) and diabetic ketoacidosis (β = 0.012; p = 0.017).

**Conclusions:**

Basal rate profiles with higher variability are associated with an increased frequency of acute complications in adults with type 1 diabetes.

## Introduction

While much attention has been attributed to the adjustment of bolus insulin doses to carbohydrate consumption, physical activity, and pre-meal blood glucose concentrations in continuous subcutaneous insulin infusion therapy (CSII), considerably less is known regarding individual basal insulin requirements.[1] However, improper basal insulin programming may cause undesirable glucose excursions between meals, under fasting conditions and during sleep as well as difficulties achieving euglycemia during exercise.[2] For example, it has recently been shown that programming CSII with increasing insulin delivery overnight to counteract the dawn phenomenon may be less effective than expected and may even increase the risk for hypoglycemia.[3] To the best of our knowledge, no studies are available that compare the effectiveness between circadian and flat basal rate programming strategies to improve glycemic control in adult type 1 diabetic patients.

The aim of this study was to identify the associations between basal rate variability and acute or chronic complications. Although basal rates and glucose profiles are intrinsically individual, identifying an association between basal insulin variation and acute or chronic complications could extend our knowledge on basal rate programming.

## Materials and Methods

### Patients

Data from the DPV-Wiss-database, a standardized, prospective, computer-based documentation of diabetes care and clinical outcomes, were analyzed. During 1995–2014, 5545 adult patients (3118 women, 2374 men) aged ≥18 years with type 1 diabetes were documented from 420 centers in Germany and Austria. Inconsistent data were reported back to the centers every 6 months for correction. Data collection was approved by the institutional review board at Ulm University and the local diabetes centers and is in accordance with the Declaration of Helsinki. Data collection in the DPV-Wiss-database is in compliance with the hospital data-protection agencies in all participating centers. Only anonymous data are transmitted for centralized analysis at the Institute of Epidemiology and Medical Biometry, University of Ulm, Ulm, Germany.

Variables evaluated were the “variability index” (VI) of basal rates, gender, age at last visit, height, weight, body mass index (defined as body weight in kilograms (kg) divided by square of height in meters), diabetes duration, total daily insulin dose, insulin dose per kg, total daily basal insulin dose, basal insulin dose per kg, prevalent diabetic retinopathy, severe hypoglycemia (defined as hypoglycemia requiring third party help), coma induced by hypoglycemia, and diabetic ketoacidosis (defined as hospital admission due to ketoacidosis with hyperglycemia >11 mmol/L and pH <7.3), prevalent microalbuminuria, and prevalent macroalbuminuria. The VI was calculated as variation of BR intervals in percent, describing the excursions of the BR profile. From each BR profile the smallest BR (> zero units) was taken as the reference BR. Next, difference in percent was calculated for each BR before and after the reference BR, added and divided by the number of BR-intervals (24 per day). Using local HbA1c reference values, HbA1c levels were mathematically standardized to the DCCT reference (range 4.05–6.05%) by means of the ‘multiple of the mean’ method to adjust for different laboratory methods.

For each patient, mean or median values of data from the most recent year of diabetes care were used for analysis. Regarding basal rate variation, the most recent basal rate was used. Following the architecture of the DPV registry, hourly basal rates (24 basal rates per day) were used for calculation. Acute (severe hypoglycemia, hypoglycemic coma and diabetic ketoacidosis) and chronic (diabetic retinopathy, micro and macro albuminuria) address a time frame of ± 6 months from the most recent basal rate.

### Statistical analysis

Calculations were done in the whole population and stratified by sex. Descriptive statistics (mean, standard deviation (SD), median, lower and upper quartile) were calculated for all variables. Spearman correlation coefficient with Fisher´s z transformation was used to calculate associations between diabetic complications, insulin doses, anthropometric measurements and BR variability. Linear regression analysis adjusted for the confounding factors age, gender, duration of diabetes, and basal rate per kg body weight was used to analyze the association between BR variability and diabetic complications. Furthermore, logistic regression models were run to estimate the association between basal rate variability, HbA1c and BMI. In regression analyses, VI was first included either as a continuous variable and subsequently categorized; F-tests or Wald tests were used to test for significance. Data from regression analysis using VI quartiles were used to construct Forest plots illustrating the odds ratio per quartile for the development of the influence on several endpoints. For all tests, a p-value of less than 0.05 was considered significant. Statistical analysis was carried out with SAS Version 9.4 (SAS Institute Inc., Cary, NC, USA) (17).

## Results

**Fig 1:**
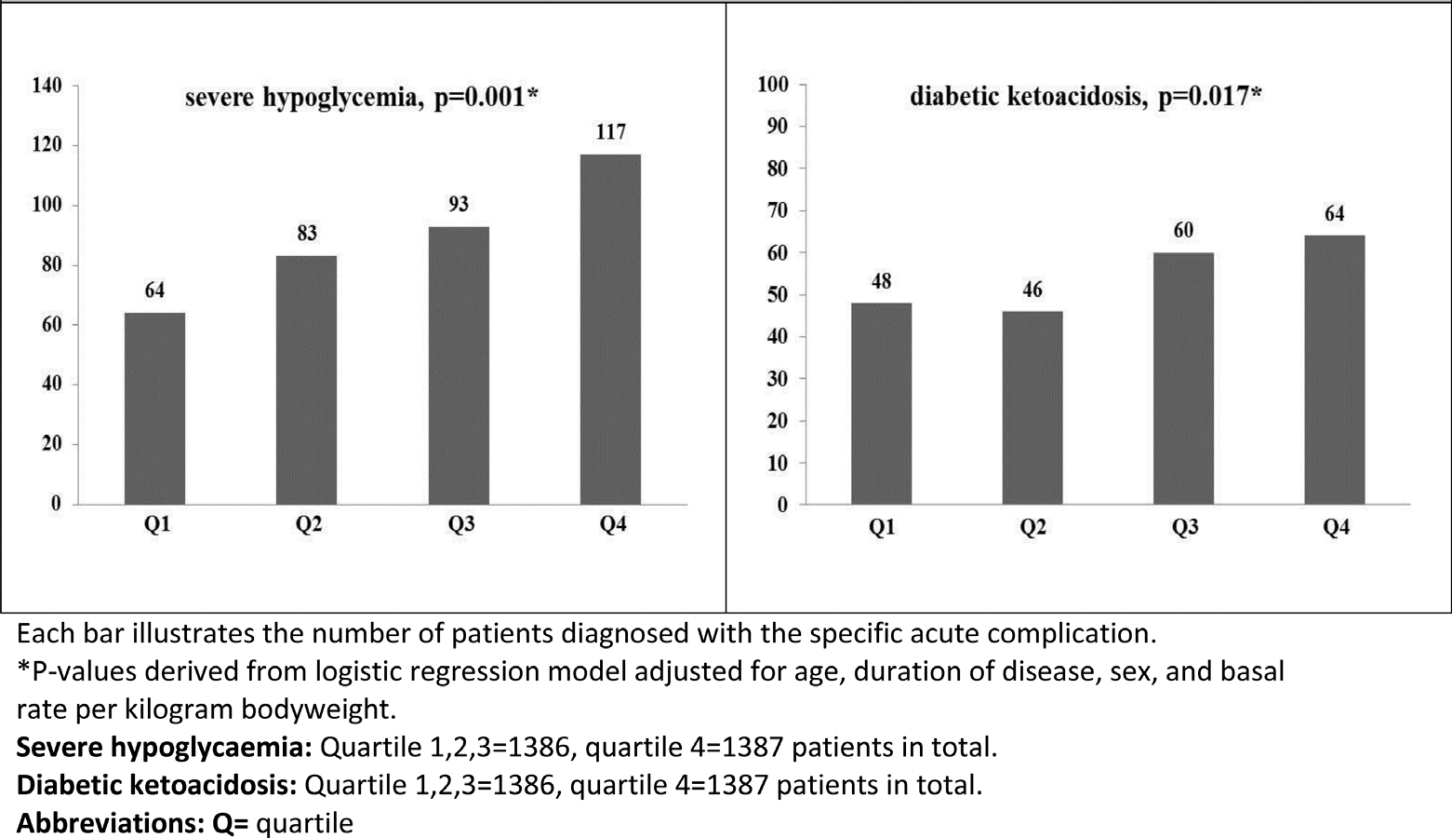
Bar graphs illustrating the number of acute complications per quartile of the variability index.

Descriptive characteristics of the study cohort are illustrated in Table 1, results of spearman rho correlations coefficient are shown in Table 2, and Table 3 illustrates results of linear regression analysis. Fig 1 illustrates the odds ratio for each VI quartile on acute complications derived from a multivariable logistic regression model. For a complete list of odds ratios for VI quartiles on several acute and chronic endpoints please refer to supplemental material (S1 Fig).

**Table 1:** Patient characteristics, separated into total cohort, women and men. N = 5545

	Total cohort, n = 5545		Women, n = 3118		Men, n = 2427	
	Mean	± STD	Mean	± STD	Mean	± STD
**Variability index (%)**	27.77	12.96	27.26	13.15	28.42	12.69
**Age (years)**	33.28	16.23	33.14	15.78	33.47	16.79
**Duration of disease (years)**	17.31	12.06	17.31	11.76	17.31	12.43
**BMI (kg/m**^**2**^**)**	25.51	4.39	25.74	4.57	25.23	4.15
**Total daily insulin dose (IU)**	71.77	30.18	66.48	28.52	78.57	30.88
**Insulin dose/kg (IU)**	0.96	0.37	0.94	0.37	0.98	0.36
**Bolus Insulin dose/kg (IU)**	0.39	0.20	0.37	0.20	0.41	0.20
**Basal Insulin dose/kg (IU)**	0.59	0.23	0.59	0.24	0.60	0.28
**Total daily BR/24h (IU)**	44.49	19.73	41.81	18.77	47.92	20.39
**BR/kg/24h (IU)**	0.59	0.23	0.59	0.24	0.60	0.23
**HbA1c (%)**	7.99	1.61	7.94	1.56	8.07	1.66
**SMBG/24h**	5.08	1.86	5.18	1.88	4.95	1.83

Data presented as mean ± standard deviation. Variability index indicates variation coefficient in basal rate intervals (%). **Abbreviations: STD =** standard deviation; **kg =** kilograms; **m =** meter; **IU =** international units; **BR =** basal rates; HbA1c = glycated hemoglobin; SMBG = self-monitoring of blood glucose

**Table 2:** Correlations of variability index with acute and chronic complications as well as patient’s characteristics in adult type 1 diabetic patients. N = 5545.

	Variability index^A^		Variability index ♀		Variability index ♂	
**Severe hypoglycemia (y/n)**^B^	**0.059**	**p = 0.002**	**0.058**	**p = 0.001**	**0.050**	**p = 0.004**
**Diabetic ketoacidosis (y/n)**	0.025	p = 0.068	0.013	p = 0.469	**0.044**	**p = 0.029**
**Hypoglycemic coma (y/n)**	**0.041**	**p<0.001**	0.032	p = 0.071	**0.058**	**p = 0.013**
**Microalbuminuria (%)**	**0.045**	**p = 0.006**	0.036	p = 0.101	0.046	p = 0.058
**Macroalbuminuria (%)**	0.015	p = 0.362	0.015	p = 0.421	0.006	p = 0.797
**Retinopathy (%)**	0.029	p = 0.135	0.031	p = 0.218	0.006	p = 0.399
**Proliferative retinopathy (%)**	0.031	p = 0.109	**0.051**	**p = 0.044**	0.006	p = 0.847
**Sex (male)**	**0.059**	**p<0.001**	n.a.	n.a.	n.a.	n.a.
**Age (years)**	**0.228**	**p<0.001**	**0.196**	**p<0.001**	**0.266**	**p<0.001**
**Duration of disease (years)**	**0.067**	**p<0.001**	**0.044**	**p = 0.013**	**0.097**	**p<0.001**
**Body weight (kg)**	0.015	p = 0.265	**-0.042**	**p = 0.021**	**0.053**	**p = 0.010**
**BMI (kg/m**^**2**^**)**	**0.056**	**p<0.001**	0.001	p = 0.938	**0.132**	**p<0.001**
**Total daily insulin dose (IU)**	**-0.223**	**p<0.001**	**-0.239**	**p<0.001**	**-0.244**	**p<0.001**
**Bolus Insulin dose/kg (IU)**	**-0.098**	**p<0.001**	**-0.069**	**p<0.001**	**-0.153**	**p<0.001**
**Basal Insulin dose/kg (IU)**	**-0.324**	**p<0.001**	**-0.323**	**p<0.001**	**-0.328**	**p<0.001**
**Insulin dose/kg (IU)**	**-0.273**	**p<0.001**	**-0.261**	**p<0.001**	**-0.298**	**p<0.001**
**Total daily BR/24h (IU)**	**-0.269**	**p<0.001**	**-0.296**	**p<0.001**	**-0.264**	**p<0.001**
**BR/kg/24h (IU)**	**-0.324**	**p<0.001**	**-0.324**	**p<0.001**	**-0.327**	**p<0.001**
**HbA1c (%)**	**-0.127**	**p<0.001**	**-0.111**	**p<0.001**	**-0.153**	**p<0.001**
**SMBG/24h**	**0.041**	**p = 0.004**	**0.043**	**p = 0.024**	**0.048**	**p = 0.029**

Variability index indicates variation in basal rate intervals (%), bold areas indicate significant correlations.

**A =** upper line illustrates spearman´s rho, lower line illustrates the test probability.

**B =** severe hypoglycemia was blood glucose concentrations <70milligrams/deciliter + requirement of outside assistance.

**Abbreviations: n.a. =** not applicable; **BR =** basal rates; **kg =** kilograms; **m =** meter; **IU =** international unit; **h =** hours; **y =** yes; **n =** no; HbA1c = glycated hemoglobin; SMBG = self-monitoring of blood glucose

**Table 3:** Multivariable logistic regression model illustrating the relationship between acute and chronic complications (defined as categorical outcome variables (yes/no)) variability index and confounders in adult type 1 diabetic patients. N = 5545.

	Severe hypoglycemia^A^	DKA	Hypoglycemic coma	HbA1c^B^	Micro-albuminuria^C^	Macro-albuminuria^D^	Retinopathy	Proliferative retinopathy
**Variability Index**	**0.013, p = 0.001**^E^	**0.013, p = 0.017**	0.008, p = 0.116	-0.002, p = 0.318	0.005, p = 0.160	-0.003, p = 0.729	-0.002, p = 0.659	<-0.001, p = 0.996
**Age (years)**	**0.014, p = 0.001**	0.008, p = 0.159	**0.022, p<0.001**	**-0.017, p<0.001**	**0.016, p<0.001**	**0.023, p = 0.010**	**0.015, p = 0.001**	0.008, p = 0.163
**Duration of disease (years)**	0.010, p = 0.065	0.001, p = 0.884	0.012, p = 0.106	0.003, p = 0.241	**0.023, p<0.001**	**0.030, p = 0.003**	**0.088, p<0.001**	**0.078, p<0.001**
**Sex (♀)**	-0.135, p = 0.227	0.205, p = 0.154	-0.069, p = 0.645	**-0.125, p = 0.003**	**-0.268, p = 0.005**	-0.232, p = 0.323	0.038, p = 0.734	0.167, p = 0.251
**BR/kg/24h**	-0.254, p = 0.334	**1.743, p<0.001**	0.119, p = 0.725	**1.545, p<0.001**	**0.932, p<0.001**	0.011, p = 0.984	0.239, p = 0.322	0.476, p = 0.121

Variability index indicates variation coefficient in basal rate intervals (%), bold areas indicate significant correlations.

**A =** severe hypoglycemia was blood glucose concentrations <70milligrams/deciliter + requirement of outside assistance.

**B =** HbA1c, corrected according to the Diabetes Control and Complications Trial (DCCT) was used for analysis

**C =** Microalbuminuria: persistent albumin excretion between 30 and 300 milligrams/24 hours

**D =** Macroalbuminuria: Albumin excretion above 300 mg/24 hours

**E =** beta-estimate and test probability, adjusted for age, sex, duration of disease and basal rates/kilogram/24 hours.

**Abbreviations: DKA =** diabetic ketoacidosis; **HbA1c =** glycated hemoglobin; **BR =** basal rates; **kg =** kilograms; **h =** hours

In total, 5545 patients including 3118 female and 2427 male adult patients entered data analysis. Mean age ± SD was 33.3 ± 16.2 years; mean duration of diabetes was 17.3 ± 12.1. Mean total insulin dose was 71.8 ± 30.2 international units (IU); mean total daily basal rate was 44.5 ± 19.7 IU. Mean prandial insulin dose was 0.39 ± 0.20 IU per kilogram (kg). Female patients used 0.37 ± 0.20 IU per kg, while male patients used 0.41 ± 0.20 IU per kg. Mean basal insulin dose per kg was 0.59 ± 0.23. Female patients used 0.59 ± 0.24 IU per kg, while male patients used 0.60 ± 0.28 IU per kg. Mean percentage of glycated hemoglobin was 7.99 ± 1.61% in the entire cohort, 7.94 ± 1.56% for female patients, and 8.07 ± 1.66% for male patients, respectively. The number of blood glucose measurements (self-monitoring of blood glucose (SMBG)) was 5.18 ± 1.88 for female patients and 4.95 ± 1.83 for male patients per day, respectively. Mean variability in basal rate was 27.8 ± 12.9% for the entire cohort, 27.24 ± 13.16% for female patients, and 28.41 ± 12.69% for male patients, respectively.

Severe hypoglycemia was diagnosed in 357 (6.4%) patients. Of these, 190 (3.4%) patients experienced hypoglycemic coma. Diabetic ketoacidosis was found in 218 (3.9%) patients. Diabetic retinopathy was found in 573 (10.4%) patients, proliferative retinopathy was found in 258 (4.6%) micro- and macroalbuminuria was diagnosed in 547 (9.9%) and 80 (1.4%) patients, respectively.

Basal rate variability was positively correlated with severe hypoglycemia (p<0.001), hypoglycemic coma (p = 0.002), and microalbuminuria (p = 0.006). In addition, VI positively correlated with age (p<0.001), duration of diabetes (p<0.0001), body height (p = 0.021), and BMI (p<0.0001), and SMBG (p = 0.004)but negatively with total daily insulin (p<0.001), insulin dose per kg (p<0.001), total daily basal rate (p<0.0001), basal rate per kg (p<0.001), and bolus dose per kg (p>0.001).

Logistic regression analysis adjusted for age, gender, duration of disease and total basal insulin confirmed significant positive correlations of VI with severe hypoglycemia (β = 0.013; p<0.001) and diabetic ketoacidosis (β = 0.012; p = 0.017).

## Discussion

The present study illustrates an association between higher individual variability in basal rates and for a higher frequency of acute complications in T1DM patients treated by CSII (Fig 1). In a cohort of approximately 5000 adult T1DM patients, higher variability in individual basal rates correlated to an increased prevalence of severe hypoglycemia and diabetic ketoacidosis, independent of total daily insulin, age, duration of disease, and gender. To the best of our knowledge the association between BR variability and the frequency of acute diabetic complications is up to date unknown.

### Which factors could explain our findings?

1) CSII allows for the programming of time-dependent insulin infusion rates. With the aim of delivering insulin in a physiological manner, allegedly to account for circadian changes in insulin-sensitivity.[4] Recently, Bouchonville et al. investigated whether programming a nightly basal rate to counteract the dawn-phenomenon was successful or not. It was found that CSII programming was neither associated with a reduction in the occurrence nor with the magnitude of the dawn-phenomenon, but rather increased the risk of hypoglycemia.[3] Though, a higher variability of the BR to counteract the dawn-phenomenon may be a possible explanation for the present results.
2) Another factor contributing to glucose variability in CSII may be insulin resorption. Absorption of subcutaneously injected insulin is known to vary by 15–30%, depending on drug quantity, subcutaneous blood flow, injection site, and composition of connective tissue.[5–7] Also, the size of the subcutaneous insulin depot may play an important role in subcutaneous pharmacokinetic.[8] Stable absorption and release of subcutaneously injected insulin has been shown to improve glycemic control. Maybe higher variability within BR causes fluctuations in insulin action potentially contributing to an increased frequency of severe hypoglycemia in this analysis. However, inappropriate basal insulin substitution correlates to recurrent and/or severe hypoglycemia as well as to chronic hyperglycemia.[2] It is debatable whether the number of patients having insufficient insulin resorption was large enough to co-create the illustrated effect of basal rate variation on acute complications. Nevertheless, constraints in insulin resorption may increase glucose variability in some T1DM patients.
3) In the present analysis, variations in basal rate patterns were associated with age, male gender, duration of disease, and body mass index (BMI). This is in line with a retrospective analysis indicating that age increased the frequency of peaks and nadirs in basal rate patterns.[1] Several studies confirm that insulin resistance is associated with the presence of chronic complications, both micro- and macrovascular, and increased mortality in T1DM patients.[9–12] Obesity and associated metabolic and hormonal traits are known promoters of insulin resistance. Whereas BMI was lower in T1DM before the Diabetes Control and Complications Trial was conducted, the percentage of overweight and obese T1DM patients is close to 50% in adults and children nowadays.[10,13,14]

Male study participants had higher variations in basal rate patterns than female, and as mentioned above, inappropriate basal rates may promote acute hypoglycemia. A recent study illustrated gender differences in glycemic control and the burden of disease between male and female T1DM patients. It was found that fear of hypoglycemia was more prevalent in women compared to men.[15] Hereby, men could be less intimidated by hypoglycemic events and therefore retain potentially inappropriate basal rates longer or more often than female T1DM patients.

4) The present results can also be interpreted inversely. As is written below, the recommended strategy to match basal CSII treatment to the patient’s requirements is to program an estimated circadian profile. If acute complications (e.g. hypoglycemic events) appear, the initial profile is adapted in a stepwise manner until ones individual insulin requirements are met. The more adaptions are necessary, the higher is the chance that the variability in one’s individual daily basal rate profile increases. Therefore, it cannot be ruled out that our results represent patients that were more difficult to treat rather than they reflect an association between BR variability and the prevalence of acute diabetic complications.
5) Another conceivable scenario is the following: A high VI could identify patients who rely on basal rate doses rather than bolus doses for the correction of glycemic variability. In this scenario, those patients’ bolus regiments are inappropriate regarding correction or carbohydrate doses, which would also lead to a higher chance for acute complications.

### What are the clinical implications of these results?

The question remains to what extend a more constant basal rate is able to prevent acute complications in T1DM. Notably, the present cross-sectional study is not able to answer the question of how much does VI increase the risk for acute complications in T1DM. In addition, there are no evidence-based recommendations available for the optimal basal rate patterns in the treatment of adult T1DM.

It is not reasonable to expect that requirements for basal insulin will be met by a single “flat rate” of insulin delivery, as circadian changes in counter-acting hormones and insulin sensitivity are significant and predictable.[1,16] Finding the presumed optimal, initial basal rate pattern is achieved by algorithms and fasting tests under controlled conditions.[17] Frequently used algorithms that estimate individual basal rate requirements comprise for example Bode’s approach, which uses up to four different basal rates per day, the constant basal rate pattern according to Walsh, and Renner´s circadian basal rate profile.[18–20] Afterwards, the optimal individual basal rate pattern is determined by stepwise titrations to address individual requirements due to physiology, diet, physical activity, and self-experience. We also calculated VI for basal rat profiles scheduled by the widely used Renner system. Renner´s approach creates a double peaked basal rate profile that is thought to mirror circadian insulin requirements. The related VI was calculated by 36.9%, which is significantly higher than the VI of the actual basal rates in the present study cohort. In clinical practice, Renner´s initial basal rate profiles have to be titrated with ongoing duration of disease in order to account for individual, actual insulin requirements.

A higher variability within BR was found in T1DM patients who experienced a higher frequency of severe hypoglycemic events. We suggest starting with a constant BR and, subsequently, to carefully adjust basal rate patterns to address individual needs depending on patient-specific requirements.

The relevance of the present study is limited by the following: The study design is only able to illustrate an association between the prevalence of complications and basal rate variability but it does not allow for the identification of causative coherences. Also, the effect size achieved in the present study does not inform about what fraction of the study population could benefit from reduced variability unless the study would indicate causation. The small effect size may not just derive from a small effect in the correlation of variability in basal rates and the frequency of acute complications, but could rather stem from a third, unmeasured variable that weakly correlates with the measured variables.

The–presumably–most important limitation is the onetime measurement of VI, which does not allow for identifying the direction, in which the variability points. In this cross-sectional design we do not know whether a higher VI would even be beneficial in the same patient, if measured prospectively. The most recent basal rate was used for analysis, including a timeframe of ± 6 months prior and after data entry. This cross-sectional design may have yielded a limited number of events (i.e. acute and chronic complications).

The strength of the study is the novelty of the findings. To the best of our knowledge this is the first study, which could demonstrate an association of acute diabetic complications and variability of BR. Another strength is the large number of patients analyzed in this study.

### What kind of attention should we pay to these results?

The present results have to be interpreted with caution. On the one hand, a very simple approach of how to measure basal rate variability correlated to the frequency of acute complications independent of numerous established confounding factors. On the other hand, the data available arise from a registry, which collects anamnestic data. Registry data only reflect an approximation of the real incidence of e.g. acute complications, depending on the quality of the patient’s event diaries, memories and quality of the anamnesis during the visit.

The optimal cohort study to investigate whether basal rate variability does in fact increase the incidence of diabetic complications would be longitudinal, randomized, and interventional. With such a design, the net effect of BR variability could be determined in a controlled fashion, though it´s not viable from an ethical point of view to withhold necessary adaptions in basal rates from a cohort.

It cannot be assumed that–following the results of the present study–a majority of T1DM patients on CSII would benefit from a flat basal rate in a way that they can avoid acute complications. First, registry data underlie the limitations described above and second, the effect size of the present results is cannot be interpreted correctly without knowledge about causation. Nevertheless, the effect was determined in a very large and well selected cohort, and was still present in linear regression after multiple adjustments. In fact, experts in T1DM should neither over-interpret nor ignore the results of the present study, as even though the study has several limitations, the correlation was present in a very large cohort, reflecting the possibility that a certain sub-cohort of T1DM patients could benefit from reduced variability in daily basal rates.

## Conclusions

In conclusion, the present cross-sectional study is the first large-scale analysis illustrating an association between higher variation in daily basal rates and the frequency of acute complications in T1DM. Higher variation in daily basal rates may identify patients who are more difficult to treat regarding glycemic goals in T1DM. A randomized trial would be necessary to determine whether this association is causal, and whether reducing the variability of basal rates can reduce acute complications in patients with T1DM.

## Supporting Information

S1 Fig Bar graphs illustrate the number of chronic complications per quartile of the variability index. (TIF)

## References

[1] Scheiner G, Boyer BA (2005) Characteristics of basal insulin requirements by age and gender in Type-1 diabetes patients using insulin pump therapy. Diabetes Res Clin Pract 69: 14–21. 15955383 10.1016/j.diabres.2004.11.005

[2] L. Fredrickson RRR, Rubin S. (2001) Optimal Pumping: A Guide to Good Health with Diabetes. Northridge (CA): MiniMed Inc.

[3] Bouchonville MF, Jaghab JJ, Duran-Valdez E, Schrader RM, Schade DS (2014) The Effectiveness and Risks of Programming an Insulin Pump to Counteract the Dawn Phenomenon in Type 1 Diabetes. Endocr Pract: 1–25. 10.4158/EP14198.OR 25100389

[4] Rubin RR, Peyrot M (2010) Patient-reported outcomes and diabetes technology: a systematic review of the literature. Pediatr Endocrinol Rev 7 Suppl 3: 405–412. 20877254

[5] Scheindlin S (2004) Transdermal drug delivery: PAST, PRESENT, FUTURE. Mol Interv 4: 308–312. 15616157 10.1124/mi.4.6.1

[6] Heinemann L (2002) Variability of insulin absorption and insulin action. Diabetes Technol Ther 4: 673–682. 12450450 10.1089/152091502320798312

[7] Lauritzen T, Binder C, Faber OK (1980) Importance of insulin absorption, subcutaneous blood flow, and residual beta-cell function in insulin therapy. Acta Paediatr Scand Suppl 283: 81–85. 7010905 10.1111/j.1651-2227.1980.tb15323.x

[8] Thomsen M, Poulsen M, Bech M, Velroyen A, Herzen J, et al (2012) Visualization of subcutaneous insulin injections by x-ray computed tomography. Phys Med Biol 57: 7191–7203. 10.1088/0031-9155/57/21/7191 23060123

[9] Kilpatrick ES, Rigby AS, Atkin SL (2007) Insulin resistance, the metabolic syndrome, and complication risk in type 1 diabetes: "double diabetes" in the Diabetes Control and Complications Trial. Diabetes Care 30: 707–712. 17327345 10.2337/dc06-1982

[10] Chillaron JJ, Goday A, Flores-Le-Roux JA, Benaiges D, Carrera MJ, et al (2009) Estimated glucose disposal rate in assessment of the metabolic syndrome and microvascular complications in patients with type 1 diabetes. J Clin Endocrinol Metab 94: 3530–3534. 10.1210/jc.2009-0960 19584183

[11] Orchard TJ, Olson JC, Erbey JR, Williams K, Forrest KY, et al (2003) Insulin resistance-related factors, but not glycemia, predict coronary artery disease in type 1 diabetes: 10-year follow-up data from the Pittsburgh Epidemiology of Diabetes Complications Study. Diabetes Care 26: 1374–1379. 12716791 10.2337/diacare.26.5.1374

[12] Yip J, Mattock MB, Morocutti A, Sethi M, Trevisan R, et al (1993) Insulin resistance in insulin-dependent diabetic patients with microalbuminuria. Lancet 342: 883–887. 8105164 10.1016/0140-6736(93)91943-g

[13] Conway B, Miller RG, Costacou T, Fried L, Kelsey S, et al (2010) Temporal patterns in overweight and obesity in Type 1 diabetes. Diabet Med 27: 398–404. 10.1111/j.1464-5491.2010.02956.x 20536510 PMC3129711

[14] van Vliet M, Van der Heyden JC, Diamant M, Von Rosenstiel IA, Schindhelm RK, et al (2010) Overweight is highly prevalent in children with type 1 diabetes and associates with cardiometabolic risk. J Pediatr 156: 923–929. 10.1016/j.jpeds.2009.12.017 20223481

[15] Anderbro T, Amsberg S, Adamson U, Bolinder J, Lins PE, et al (2010) Fear of hypoglycaemia in adults with Type 1 diabetes. Diabet Med 27: 1151–1158. 10.1111/j.1464-5491.2010.03078.x 20854383

[16] Beyer J, Krause U, Dobronz A, Fuchs B, Delver JR, et al (1990) Assessment of insulin needs in insulin-dependent diabetics and healthy volunteers under fasting conditions. Horm Metab Res Suppl 24: 71–77. 2272630

[17] J. Walsh RR (2000) Pumping Insulin: Everything You Need for Success with an Insulin Pump. San Diego: Torrey Pines Press.

[18] Bode BW (1995) Establishing and verifying basal rates In: FREDRICKSON L, ed. The Insulin Pump Book: Insights from the Experts. 1995 ed. Los Angeles: MiniMed Inc. pp. 48–58.

[19] Walsh JRR (2006) Pumping Insulin: Everything You Need for Success on a Smart Insulin Pump. San Diego: Torrey Pines Press.

[20] Wizemann E (2001) Prospective evaluation of a standardized basal rate distribution for CSII in type 1 diabetes over 6 months. Diabetologie und Stoffwechsel 10.

