# Supplementary figures and images for: Variability of Basal Rate Profiles in Insulin Pump Therapy and Association with Complications in Type 1 Diabetes Mellitus
Source: PLoS One. 2016 Mar 3;11(3):e0150604. doi: 10.1371/journal.pone.0150604 (PMC4777503; doi:10.1371/journal.pone.0150604)

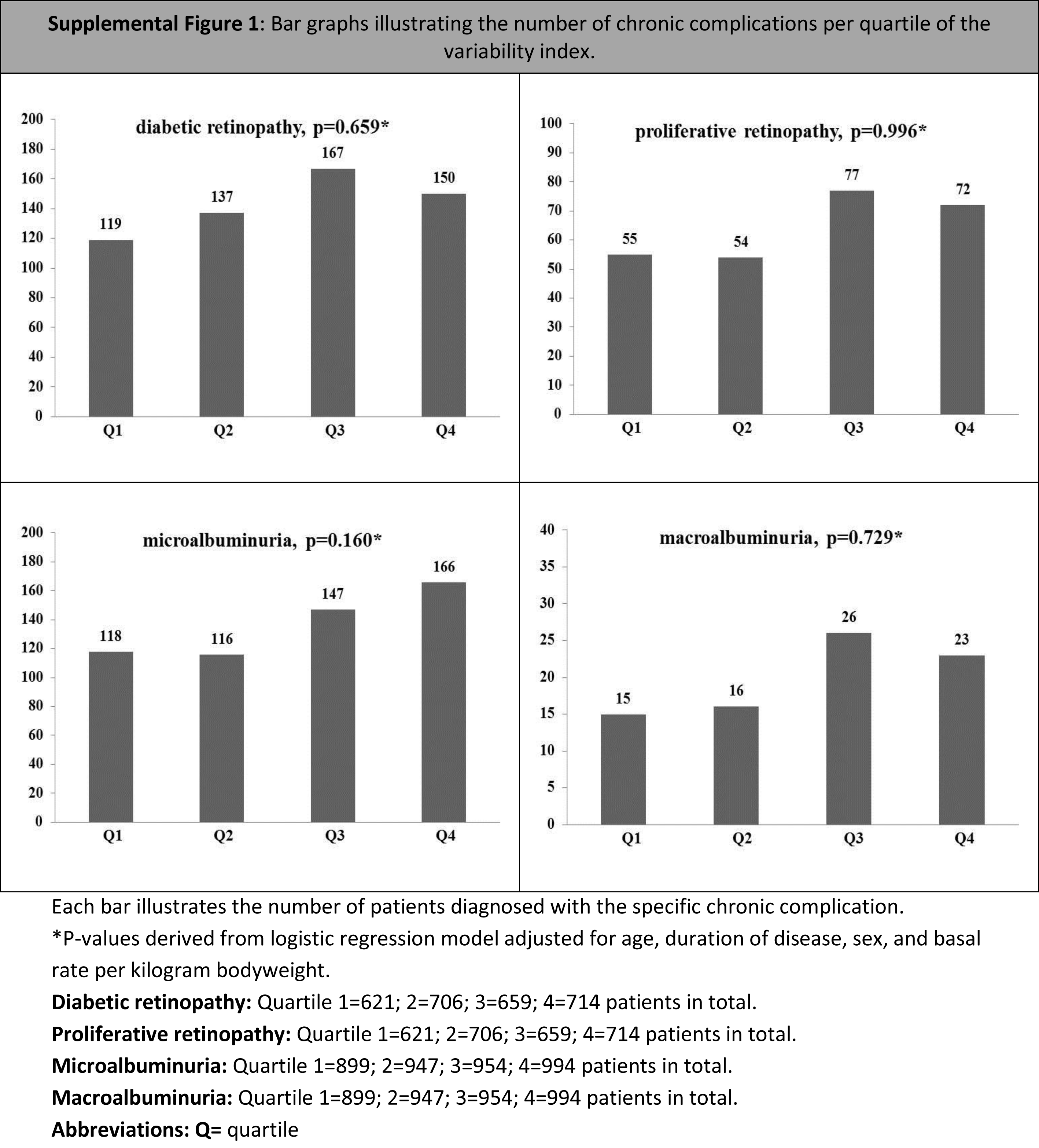

Supplement: S1 Fig — (TIF) [file pone.0150604.s001.tif]
